# Axillary brachial plexus blockade in moyamoya disease?

**DOI:** 10.4103/0019-5049.79897

**Published:** 2011

**Authors:** Saban Yalcin, Hasan Cece, Halil Nacar, Mahmut Alp Karahan

**Affiliations:** Department of Anesthesiology, Reanimation, Harran University Medical Faculty, Şanliurfa, Turkey; 1 Department of Radiology, Harran University Medical Faculty, Şanliurfa, Turkey

**Keywords:** Brachial plexus block, moyamoya disease, orthopaedic surgery

## Abstract

Moyamoya disease is characterized by steno-occlusive changes of the intracranial internal carotid arteries. Cerebral blood flow and metabolism are strictly impaired. The goal in perioperative anaesthetic management is to preserve the stability between oxygen supply and demand in the brain. Peripheral nerve blockade allows excellent neurological status monitoring and maintains haemodynamic stability which is very important in this patient group. Herein, we present an axillary brachial plexus blockade in a moyamoya patient operated for radius fracture.

## INTRODUCTION

Moyamoya disease (MMD) is characterized by steno-occlusive changes at the terminal portions of the intracranial internal carotid arteries with compensatory development of a hazy network of small collateral vessels at the base of the brain. These abnormal vessels look like a puff of cigarette smoke in angiography, which is described as “moyamoya” in Japanese.[1,2]

Cerebral blood flow and metabolism are strictly impaired in most cases of MMD. Perioperative stroke occasionally occurs after the surgical procedure.[3] Hence anaesthetic management is very important in this patient group. The goal in perioperative management is to preserve the stability between oxygen supply and demand in the brain. Hypocapnia, hypercapnia, hypotension and hypovolemia during surgery have all been identified as risk factors for ischaemic complications.[4,5]

According to our literature review both general[6,7] and neuraxial[2] anaesthesia have been used successfully in this patient group; however, there were no reports about peripheral nerve blocks. This is the first case, reporting axillary brachial plexus blockade in a MMD patient who was operated for radius fracture.

## CASE REPORT

**Figure 1:**
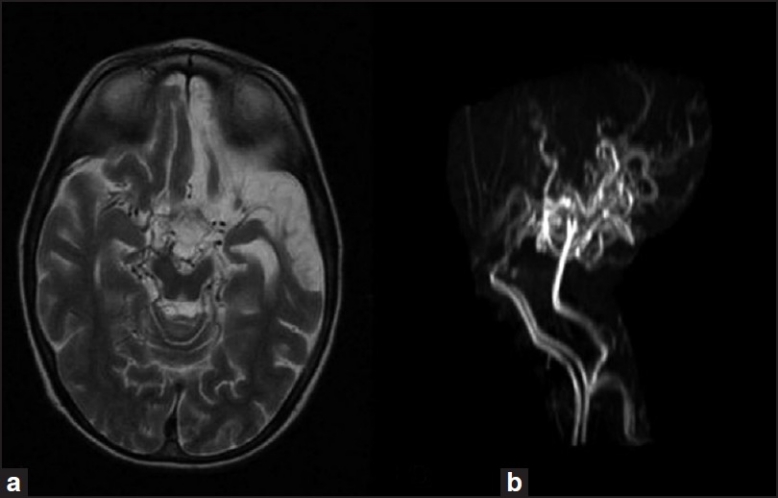
(a) Axial T2WI MRI shows curvilinear “net-like” filling defects within the ambient cistern corresponding to collateral moyamoya vessels and (b) MRA shows occlusion of both distal ICAs, non-visualization of MCAs and ACAs with collateral lenticulostriate vessels. MRI, magnetic resonance imaging; MRA, magnetic resonance angiography; ICAs, internal carotid arteries; MCAs, middle cerebral arteries; ACAs, anterior cerebral arteries

A 15-year-old patient was referred to our hospital for the surgical treatment of his right radius fracture 3 years ago. He presented with problems of transient paralysis in his right arm and both legs. He was diagnosed as suffering from MMD according to the magnetic resonance imaging and magnetic resonance angiography [Figure 1], and medical treatment was started. He sustained fracture of the right radius in a motor vehicle accident and surgery was planned for his right radius. On physical examination there were no abnormalities except tenderness in his right arm. Laboratory studies revealed no abnormalities. He was using 100 mg acetylsalycilic acid once in a day for MMD and no ischaemic attacks were seen in the last 1 year. The patients’ weight was 47 kg. After arrival in the operating room, an 18-gauge intravenous catheter was placed in the forearm contralateral to fracture; premedication was given intravenously (0.03 mg/kg midazolam). Standard monitoring was used throughout the procedure, including noninvasive arterial blood pressure, heart rate and pulse oximetry. The patient was placed in the supine position with the arm abducted to approximately 90Ŷ with the hand resting on a pillow next to the head. The nerve location was performed with the aid of a nerve stimulator (braun stimuplex HNS 11 peripheral nerve stimulator) and 22-gauge 5 cm-long, short-beveled, teflon-coated needles (braun stimuplex A needle). The nerve stimulator was set with a pulse duration of 0.15 ms, a current intensity of 1 mA and a frequency of 2 Hz. It has been suggested that the axillary brachial plexus sheath contains septae preventing local anaesthetic from reaching all neuronal components contained within the sheath. The clinical significance of these septae remains controversial but we tried to locate all four main branches (radial, musculocutaneous, median, ulnar) and blocked them separately with 4 ml of 0.5 % levobupivacaine each. The musculocutaneous nerve leaves the brachial plexus sheath proximally. Owing to the large area covered by this nerve and its importance in achieving complete anaesthesia, we also paid attention to achieve specific twitches of this nerve (biceps twitch, arm flexion). The other nerves were located according to the specific twitches elicited by their stimulation: Radial nerve: Arm and finger extension, supination; median nerve: Wrist, second and third finger flexion, pronation; ulnar nerve: Fourth and fifth finger flexion, thumb adduction. After the proper twitch was elicited, the stimulating intensity was progressively reduced to less than 0.5 mA maintaining the proper twitch; then, 1 ml local anaesthetic was injected. After this injection stopped the twitch, the location was considered adequate, and the remaining 3 ml was injected. Then, the needle was withdrawn to the skin and redirected, looking for the next twitch. 16 ml 0.5% levobupivacaine was used totally. Sensory block was assessed as loss of pinprick sensation in the central sensory region of each of the four nerves with the same stimulus delivered to the contra lateral side. Motor block was evaluated using arm and wrist flexion/extension for median and musculocutaneous nerve, finger extension for radial nerve, and thumb and fifth digit pinch for ulnar nerve. Patient was ready for surgery in 20 minutes. The surgery performed on his right radius was open reduction and internal fixation which lasted 55 minutes. The blood pressure was well controlled and there were no need for additional intravenous agents for pain during the operation. The patient was discharged in two days.

## DISCUSSION

This case report focuses on the anaesthetic management of this rare MMD in an emergency situation and remarks the significance of adequate anamnesis and preoperative evaluation and reports a successful peripheral nerve blockade anaesthesia in an MMD patient.

MMD is a chronic occlusive cerebrovascular disorder of unknown aetiology. The highest incidence of MMD is in the first decade of life. Onset in childhood is often marked by ischaemic symptoms, whereas intracranial haemorrhage is more typical in adults. Increased familial incidence has been reported. Responsible genes were reported to be located in chromosomes 3, 6 and 17.[5,8] Revascularization surgery, such as superficial temporal artery-middle cerebral artery bypass and encephaloduroarteriosynagiosis, is the traditional treatment for ischaemic attacks. Whether this palliative surgery lessens future haemorrhagic events, is still arguable. Medical treatment with vasodilators, corticosteroids, antiplatelet agents, etc. has been tried with doubtful efficacy.[8]

During pre-anaesthesia evaluation of a patient with MMD, particular concentration should be paid to neurological status, frequency of ischaemic attacks, CT and/or MRI evidence of infarction and angiographic signs of low perfusion or cerebrovascular reactivity.[4] The principles of anaesthetic management for patients with MMD are to maintain normotension, normovolemia, normocapnia, and normothermia to avoid the cerebral ischaemia because of restricted cerebrovascular reserve capacity.[9] There were several reports about MMD patients who underwent operations with general anaesthesia and neuroaxial anaesthesia. Most reports about general anaesthesia are reported in revascularization surgery for MMD. During general anaesthesia, hyperventilation and the resultant hypocapnia may reduce cerebral blood flow and thus precipitate ischaemic symptoms. Not only hypocapnia but also hypercapnia during the operation increases the risk of perioperative stroke because hypercapnia sometimes induces the steal phenomenon of the regional cerebral blood flow.[3,10,11] Volatile anaesthetics and nitrous oxide can cause cerebral vasodilation, and this may also result in intracerebral steal. Propofol, etomidate and thiopental are all known to decrease both the cerebral metabolic rate for oxygen and cerebral blood flow in dose dependent fashion. As these drugs are cerebral vasoconstrictors, there is no danger of steal phenomenon occurring.[5] Kikuta *et al* believed that intravenous anaesthesia with propofol has potential to provide brain protection in surgery for MMD.[10] Balanced anaesthesia and total intravenous anaesthesia are considered the most suitable choices for revascularization procedures in MMD.[5,12]

Neuraxial anaesthesia reports in MMD are especially focused on obstetric anaesthesia. Profits of neuraxial anaesthesia over general anaesthesia are reported to be as follows (1) it is easy to assess neurological changes which are essential in MMD, and (2) neuraxial anaesthesia avoids hypertension which often results from tracheal intubation and extubation. Shortcomings are (1) hypotension is more likely, and (2) patients might develop hyperventilation and hypertension due to anxiety.[2] Kato *et al*. believed that potential disadvantages of neuraxial anaesthesia, hypotension and anxiety, were treatable; hypotension could be easily managed with vasopressors and intravenous fluid and anxiety could be reduced by active use of sedatives.[2]

Anaesthetic management of emergency cases are challenging due to underlying situation. Rare disorders are not rare, if you encounter in an operating room as an emergency. It is difficult to receive comprehensive anamnesis in such cases although medical history should be the cornerstone of the anaesthetic management. In our case, we chose axillary brachial plexus blockade in order to avoid the above-mentioned undesired effects of general anaesthesia and to assess neurological status during surgery. Peripheral nerve blockade allows excellent neurological status monitoring and maintains haemodynamic stability which is very important in this patient group. On the other hand these techniques necessitate skill and would be not suitable for every operation especially in emergent cases. This is the first case to be reported, in the literature, using peripheral nerve blockade in MMD.

In conclusion, receiving comprehensive anamnesis care is significant even in emergency cases and peripheral nerve blockade anaesthesia with appropriate sedation protocols should be kept in mind for proper interventions in patients with MMD.
